# The elusive MAESTRO gene: Its human reproductive tissue-specific expression pattern

**DOI:** 10.1371/journal.pone.0174873

**Published:** 2017-04-13

**Authors:** Shlomit Kenigsberg, Patricia D. A. Lima, Leila Maghen, Brandon A. Wyse, Chantal Lackan, Annie N. Y. Cheung, Benjamin K. Tsang, Clifford L. Librach

**Affiliations:** 1 CReATe Fertility Centre, Toronto, Canada; 2 Department of Obstetrics & Gynecology, University of Ottawa, Ottawa, Canada; 3 Department of Pathology, The University of Hong Kong, Hong Kong, People’s Republic of China; 4 State Key Laboratory of Quality Research in Chinese Medicine, Macau Institute for Applied Research in Medicine and Health, Macau University of Science and Technology, Macau, China; 5 Department of Cellular & Molecular Medicine, University of Ottawa, Ottawa, Canada; 6 Interdisciplinary School of Health Sciences, University of Ottawa, Ottawa, Canada; 7 Chronic Disease Program, Ottawa Hospital Research Institute, Ottawa, Canada; 8 Department of Obstetrics and Gynecology, University of Toronto, Toronto, Canada; 9 Department of Gynecology, Women’s College Hospital, Toronto, Ontario, Canada; Universite du Quebec a Trois-Rivieres, CANADA

## Abstract

The encoded transcript of the Maestro—*Male-specific Transcription in the developing Reproductive Organs* (MRO) gene exhibits sexual dimorphic expression during murine gonadal development. The gene has no homology to any known gene and its expression pattern, protein function or structure are still unknown. Previously, studying gene expression in human ovarian cumulus cells, we found increased expression of *MRO* in lean-type Polycystic Ovarian Syndrome (PCOS) subjects, as compared to controls. In this study, we examined the *MRO* splice variants and protein expression pattern in various human tissues and cells. We found a differential expression pattern of the *MRO* 5’-UTR region in luteinized granulosa-cumulus cells and in testicular tissues as compared to non-gonadal tissues. Our study also shows a punctate nuclear expression pattern and disperse cytoplasmic expression pattern of the MRO protein in human granulosa-cumulus cells and in testicular germ cells, which was later validated by western blotting. The tentative and unique features of the protein hampered our efforts to gain more insight about this elusive protein. A better understanding of the tissue-specific *MRO* isoforms expression patterns and the unique structure of the protein may provide important insights into the function of this gene and possibly to the pathophysiology of PCOS.

## Introduction

Granulosa cells (GCs) are cuboidal cells surrounding the oocyte in developing ovarian follicles. As a follicle matures, the GCs proliferate to form multicellular layers and those directly surrounding the oocyte form the cumulus oophorous complex (COC) [1]. Before ovulation, GCs are the primary site of estrogen production; these cells become granulosa lutein cells which produce primarily progesterone after ovulation. GCs can only be collected during ovum retrieval from patients undergoing controlled ovarian stimulation for in-vitro fertilization (IVF) and have been used extensively in studies to improve our understanding of gene function and regulation in human fertility and the pathobiology of infertility. The *Male-specific Transcription in the developing Reproductive Organ-MAESTRO* (*MRO*) gene transcript was first described in murine male gonadal development but not in female gonads [2]. The human *MRO* gene (gene ID: 83876) [3, 4] was first reported in a study comparing gene expression patterns in human ovarian cumulus cells (CCs) from lean and obese-type polycystic ovary syndrome (PCOS) versus median Body Mass Index (BMI) matched non-PCOS controls [5]. *MRO* had a 10-fold increased expression in lean-PCOS as compared to controls, together with proteoglycan 1 secretory granule (*PRG1)*; ryanodine receptor 3 (*RYR3)*; lectin galactoside-galectin 12 (*LGALS12)*; Hyaluronan and proteoglycan link protein 1 (*HAPLN1)*; chemokine (C-C motif) ligand 20 (*CCL20)*; solute carrier family 7 member 2 (*SLC7A2)*; and wingless-type MMTV integration site family, member 5A (*WNT5A*). The lack of information on the *MRO* transcript and protein, both in murine model and human, has encouraged us to further characterize the gene splice variants, tissue distribution and protein product(s). The protein structure and biological function remain to be clearly defined.

*MRO* belongs to a relatively new gene family, named ‘maestro heat-like repeat family (*MRO*H) [6]. The *MROH* family include 11 members so far (MRO, *MRO*H, *MRO*H2A, *MRO*H2B, *MRO*H3P, *MRO*H4P, *MRO*H5, *MRO*H6, *MRO*H7, *MRO*H8, *MRO*H9) and all contain HEAT repeats motif or regions that are highly similar to HEAT repeats [3]. The protein structure and function of these *MRO*H family members is also unknown. The HEAT domain, which are found in several cytoplasmic proteins including the four that give rise to the acronym HEAT [7]. Other examples for HEAT-containing proteins include the nuclear cargo transport protein Ran-GTP binding importin beta (Karyopherin, *KPNB1*) and Exportin 1 (*XPO1*) cargo transport proteins [8, 9]. This domain is structurally related to armadillo repeats (ARM), which form rod-like helical structures and are involved in intracellular transport.

**Fig 1:**
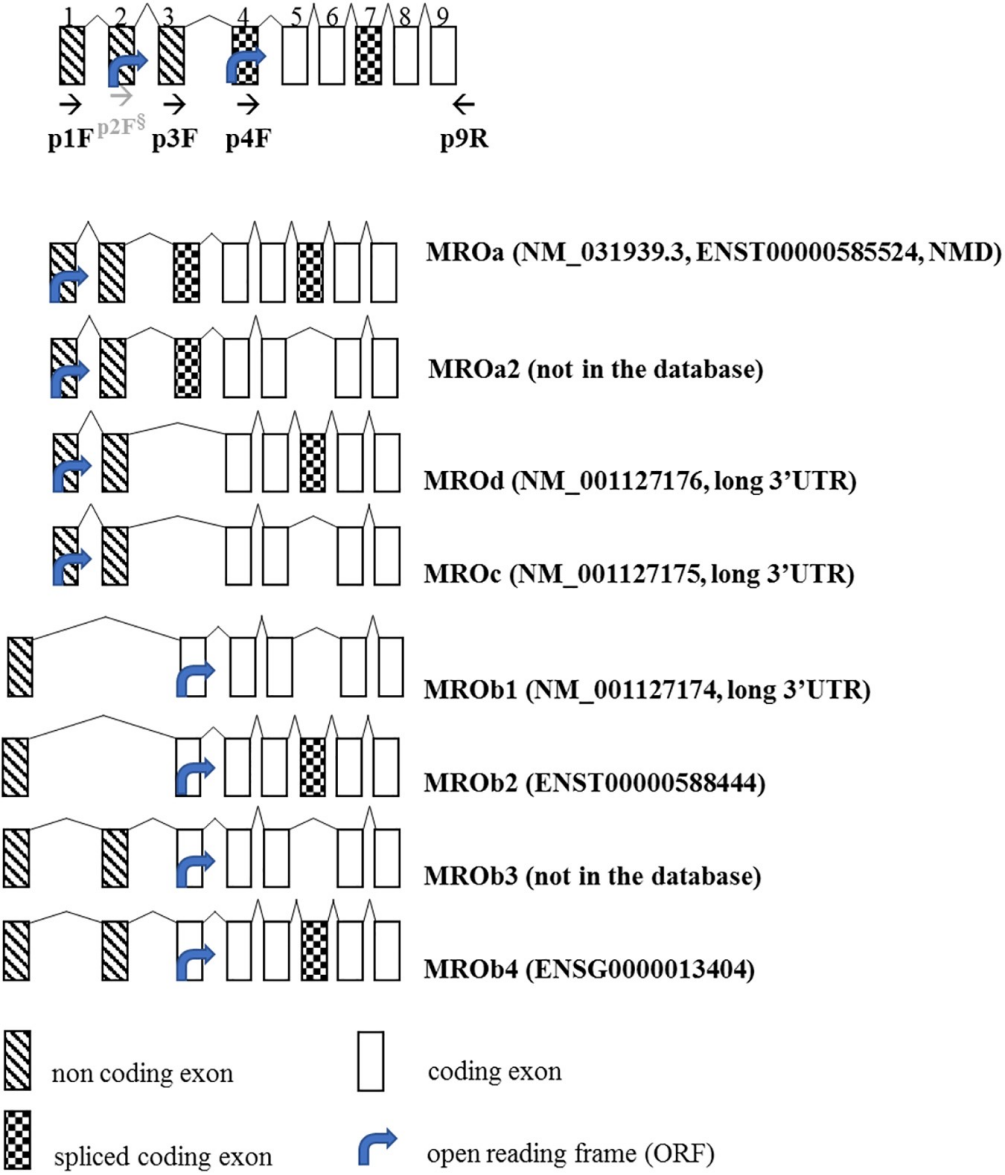
Schematic representation of the human *Maestro (MRO)* gene structure. The start codon of the Open Reading Frame (ORF) indicated by an arrow is localized in exon 2 for *MRO*a, *MRO*a2, *MRO*c, *MRO*d, and in exon 4 for ‘*MRO*b’ (b1-b4) transcript variants. *MRO*a (NM_031939.3), *MRO*d (NM_001127176.1), and *MRO*c (NM_001127175.1) coding regions consist of 753bp, 648bp, and 492bp and encode 248aa, 262aa, and 210aa protein, respectively. *MRO*b1 (NM_001127174), *MRO*b2, and *MRO*b3 consist of 678bp, 827bp, and 792bp and encode 196aa, 248aa, and 210aa proteins, respectively. *MRO*a is considered as a non-sense mediated decay (NMD) transcript. The ‘a’ variant differs by a 103bp additional 5’ exon (exon 4) compared to the ‘c’ and ‘d’ variants. The ‘c’ variant lacks one in-frame exon (exon 7) compared to ‘d’. The *MRO* ‘b’ variants (*MRO*b1-4) contain a distinct 5' UTR differ from *MRO*a, c, d by a 76bp non-coding exon (exon 1), and differ from each other by one alternate exon (exon 3) and one in-frame exon (exon 7). *MRO*b4 (BC029860.1) is the longest transcript, consisting of 948bp and encoding 248aa. The predictive protein isoforms have 2 distinct N-terminus with expected sizes of 196 aa– 248 aa and a mass of 23-29kDda (S1 Fig).

The eight deduced spliced transcripts of the novel *MRO* gene (see Fig 1) described here can give rise to four protein isoforms ranging 26–29 kDa (S1 Fig) as well as several non-sense mediated decay transcripts (NMD; NCBI [4], Ensembl [10]). *MRO* gene and protein is conserved in mammalian only (magnorder *Boreoeutheria****)*** and have no homology to other genes or proteins in the genome databases *(HomoloGene*:*41729*, [4]).

A mouse knockout model has been created and is both viable and fertile [11] however, no detailed studies have been reported on adult female mice or human *MRO* expression and cellular localization. Given the paucity of information regarding the human *MRO* gene, and its potential role in folliculogenesis, our aim was to gain a detailed understanding of *MRO* transcription patterns and protein expression in humans, with particular emphasis on the gonads.

## Materials and methods

### Isolation of human granulosa cells and cumulus cells

This study was vetted by the University of Toronto Research Ethics Board and specifically approved the use of these materials for this study (Approval #29237). With written informed consent, CCs and GCs were isolated from follicular fluid (FF) collected during standard IVF from healthy young ovum donors (n = 9) and PCOS (n = 9) patients undergoing standard IVF procedures at the CReATe Fertility Centre, Toronto, ON, Canada. PCOS was diagnosed according to the Rotterdam criteria [12]. Patients age, median body mass index (BMI), number of retrieved oocytes and hormonal profile are given in Table 1. Controlled ovarian stimulation was achieved by flexible-start antagonist protocol and underwent transvaginal ultrasound assisted follicle aspiration 36 h after triggering with human chorionic gonadotropin (hCG) and/or Gonadotropin-releasing hormone (GnRH), agonist, as described previously [13].

**Table 1:** Clinical parameters of PCOS and control patients.

	PCOS (N = 9)	Range	Controls (N = 9)	Range
**Age (years)**	26	26–36	32	30–36
**BMI (kg/m2)**	22	18 to 24	23	19.7–24.1
**Number of eggs retrieved**	24	14–28	18	5–24
**Number of follicles on cycle day 2**	>12	n/a	<10	n/a
**AMH (pmol/L)**	54.3	50.8–121.7	31.9	17.1–37.8
**E2 (pg/mL)**	468.2	236–2576	169	35.3–882
**LH (mIU/mL)**	9.41	1.0–10.0	11.27	1.0–10.0
**FSH (mIU/mL)**	5.64	2.6–8.9	5.66	2.4–14.3
**PRG (ng/mL)**	2.3	0.8–22.1	2	0.4–22.2
**Free Testosterone (pmol/L)**	7.4	0.49–9.8	1	0.49–11.4*
**Prolactin (ng/mL)**	7.7	6.5–17.4	11.1	7.8–14.2
**DHEAS (umol/L)**	3.8	2.6–10.9	6.7	4–11.8
**TSH (IU/mL)**	2.7	1.2–4.3	1	0.99–1.6
**Androstenedione (nmol/L)**	7.3	4.4–15.2	5	3.6–7.6
**17-OH (prog nmol/L)**	5.3	4.7–8.8	4.2	2.2–4.9

Median body mass index (BMI), Anti-mullerian hormone (AMH), Luteinizing hormone (LH), Follicle-stimulating hormone (FSH), Dehydroepiandrosterone sulfate (DHEAS), Thyroid stimulating hormone (TSH). Values are expressed as Median.

*outlier.

The oocyte and FF are collected from the patient by inserting an aspiration needle into the follicle under transvaginal ultrasound guidance and applying gentle suction to drain the follicle of its contents. The oocyst is taken by an embryologist and the remaining discarded material including the FF (containing GCs) were pooled. GCs were purified according to previously published methods [13–15]. Briefly, the GCs were pelleted by centrifugation (700 x g, 10 min, 4°C). The top cellular layer was collected and transferred to a new tube and was washed in a 1:1 mixture of Dulbecco’s Modified Eagle Medium and Ham’s Mixture F-12 (DMEM/F-12) (ThermoFisher Scientific, Burlington, ON, Canada), layered over equal volume of a 100% Ficoll gradient solution (GE Healthcare, Mississauga, ON, Canada) and centrifuged (700xg, 20 min, 4°C). The cellular layer at the Ficoll/PBS interface was aspirated and washed in 1x PBS. To further eliminate contaminating blood cells, the suspension was incubated (3 min, RT) with 2x Lysis-EZ buffer (BioBasic, Markham, Canada). A small portion of cells was kept aside to use as control for cell purity. After centrifugation (700xg, 10 min, 4°C), the cells pellet was washed twice with DMEM/F12 before being cultured or used for RNA and/or protein extraction. Viable cell-counts for the purified GCs were performed using the semi-automated Countess instrument (ThermoFisher) according to the manufacturer’s instruction. CCs were collected by standard mechanical microdissection of oocytes by embryologists as previously described [16, 17].

### RNA extraction and RT-PCR

Total RNA was isolated from GCs and CCs using the RNeasy Mini kit (Qiagen Inc., Toronto, ON, Canada). TURBO DNA-free kit (ThermoFisher) was used to ensure removal of genomic DNA. Commercial total RNA from human tissues (testes—pooled from 39 Caucasians, ages: 14–64; liver—Caucasian male, age: 51; kidney—Caucasian female, age: 40; ovary—pooled from 16 Caucasian, ages: 20–60) (Takara Bio USA Inc., Mountain View, CA, USA) and H68 cell line (Life Technologies, Carlsbad, CA, USA). The cDNA was prepared from 1 ug of total RNA using the QuantiTect Reverse Transcription Kit (Qiagen). PCR amplification was performed with the QuickLoad Taq enzyme mix (New England Biolabs (NEB), Ipswich, MA, USA), using the Biometra thermocycler (Biometra, Goettingen, Germany). To clone the *MRO* splice variants, 4 primer pairs were designed (Table 2) using Primer3 software [18]. PCR products were resolved on 2% agarose gels and visualized on the MiniBis gel documentation system (DNR, Kiryat-Anavim, Israel).

**Table 2:** List of PCR primers.

Primer Name	primer sequence	Expected fragment size (bp)	Annealing temp. (C°)
**p1F**	F: CACCCTTCATGAGGATGAGC	677, 955	61
**p3/5F**	F: CCGCAGGTCTCTTGGAAAC	513, 669	63
**p4F**	F: TGGACCAAAGACAGAGGAGAATC	606, 762	63
**p9R**	R: CCTGCTGCTCTGCGCTTAC		
**hACTB**	F: ATGCAGAAGGAGATCACTGC	508	55
	R: GTCCTCGGCCACATTGTGAA		

To detect and clone all potential human *MRO* splice variants, primer pairs were designed based on sequences retrieved from electronic gene databases (NM_031939.3, NM_001127174.1, NM_001127175.1, NM_001127176.1). Specific primer pair combinations (F: Forward primer, R-reverse primer) were used to characterize potential splice variants. Primers p1-Forward (p1F) together with p9-Reverse (p9-R) enabled us to clone the '*MROb*' isoforms containing the distal 5’UTR, while primer p3/5-F spanning exons 3/5, skipping exon 4, together with p9-R facilitated the cloning of *MROa*, *MROc* and *MROd* containing the proximal 5’UTR. Primer p4-Forward (p4F) together with p9-R enable the cloning of the coding region. hACTB—Human beta-actin.

The PCR products were cloned into Cloning into pGEM-Teasy vector (S1 File) and successful ligation was confirmed by sequencing at the Centre of Applied Genomics–TCAG (Hospital for Sick Children, Toronto, ON, Canada). Sequencing files can be found in Kenigsberg, Shlomit (2017): MRO clones sequence files. Figshare. https://doi.org/10.6084/m9.figshare.4779763.v1

### Quantitative PCR

For quantitative analysis, a duplex PCR (qPCR) reaction was performed using MRO TaqMan® MGB probes (Table 3) labeled with 5’-FAM reporter dye and the internal control gene probe, 18S RNA, labeled with the 5’-VIC reporter (ThermoFisher), using TaqMan® Multiplex Master Mix.

**Table 3:** List of commercial TaqMan probes used for qPCR (Thermo Fisher). The amplification program was as follows: a uracil-DNA glycosylase (UDG) incubation (50°C, 2 min) and initial DNA polymerase enzyme activation (95°C, 10 min), followed by 40 cycles of denaturation (95°C, 15 sec) and annealing ⁄ extension (60°C, 1 min).

Probe name	Exons span	Cat. #
**pQ-1/3**	exons 1, 3	Hs00901130_m1
**pQ-2/3**	exons 2, 3	Hs00903498_g
**pQ-8/9**	exons 8, 9	Hs00901134_m1
**18sRNA**		Hs99999901_s1

(ThermoFisher) on the Rotor-Gene 6000 thermocycler (Qiagen). All probes were individually validated in preliminary experiments using plasmids for each MRO clone to ensure specific, efficient, and linear amplification (data not shown). Assays were performed in triplicate and relative levels of gene expression were calculated using Rotor-Gene 6.0 software (Qiagen) and normalized to levels of 18S RNA.

### Statistics

Results were generated from GCs and CCs from 18 independent patients, each sample was run in triplicates. Relative mRNA values were expressed as Mean ± SEM. One-way ANOVA was performed using GraphPad Prism version 5.00 for Windows (GraphPad Software, San Diego California USA, www.graphpad.com). Differences were considered significant at p<0.05.

### Immuno-florescence cytochemistry (IF-ICC) on GCs and CCs in culture

GCs and CCs were cultured for 24 h in Millicell EZ 8-well glass slides (Millipore, Etobicoke, ON, Canada) in DMEM/F-12 medium (ThermoFisher) supplemented with 10% FBS and 1X Antibiotic-Antimycotic (ThermoFisher). Cells were fixed in 4% paraformaldehyde (15 min, RT) followed by permeabilization with 0.1% Triton X-100 (10 min, RT). The slides incubated with 1%BSA/5% goat serum for 1h to reduce non-specific binding, following by overnight incubation (0.4 ug/ml, 4°C) with the rabbit anti-human MRO-FIL antibody, raised against amino acid 50–150 (CAT# ab181048 from Abcam, Toronto, ON, Canada), or the FL-248 antibody, raised against the full length protein produced in *E*.*coli* (CAT# SC-134943, from Santa Cruz Biotechnology (SCBT), Dallas, Texas, USA). Slides were then incubated with FITC-conjugated goat anti-rabbit IgG secondary antibody (1:10,000; BD Biosciences, Mississauga, ON, Canada). Nuclei were counterstained with DAPI (2 μg/ml, 2 min, ThermoFisher). The immune stained cells were visualized and imaged using the EVOS fluorescence microscope (AMG, Bothell, WA, USA). Wheat germ expressed recombinant *MRO* protein [2 ug/ml (5X concentration of antibody; CAT# ab164453, Abcam)] was used as a blocking peptide to demonstrate antibody specificity, according to the manufacturer instructions. Detailed analysis of the available MRO primary antibodies and recombinant proteins used in this study is provided in S1 File, S1 and S2 Tables.

### Immuno-florescence histochemistry (IF-IHC) with human tissue sections

Immunolocalization of *MRO* was performed on 5um sections of archived formalin fixed paraffin embedded human PCOS and non-PCOS ovarian tissues after clinical and pathological investigation (Department of Pathology, Queen Mary Hospital, The University of Hong Kong). The use of these clinical materials was approved by the Institutional Review Board of The University of Hong Kong/Hospital Authority Hong Kong West Cluster (IRB reference number: UW 14–169). Briefly, tissue sections were deparaffinized and rehydrated with xylene and a serial ethanol gradient, and subjected to heat-mediated antigen retrieval in citrate buffer (10 mM, pH 6.0; 20 min, 95°C). Non-specific binding was blocked using 5% milk diluted in PBS-T (40 min, RT), following overnight incubation at 4°C with the FL-248 antibody (4 ug/ml). After washing, sections were incubated with goat anti-rabbit IgG conjugated with Alexa-fluor 488 (1h, RT), mounted in prolong gold anti-fade reagent with DAPI (ThermoFisher) and visualized using an Axioplan 2 Imaging immunofluorescence microscope with images captured and analyzed using Axion-Vision 4.8 software (Zeiss, North York, ON, Canada).

*MRO* protein expression in other human tissues was examined by IHC on tissue array microslides (TAM) from pre-menopausal human ovaries (CAT# OV801), ovarian carcinoma (CAT# T112a), testis carcinoma (CAT# TE482) and multiple organs–cerebellum section of the brain (CAT# BN501) (US BioMax, Rockville, MD, USA) at the Centre for Modeling Human Disease (Toronto, ON, Canada) as described previously [19]. Briefly, TMA sections were deparaffinized and rehydrated in a series of xylene and ethanol washes, incubated with H_2_O_2_ (0.3%; 30 min) to quench endogenous peroxidases and subjected to antigen retrieval with the Trilogy system (Cell-Marque, Rocklin, CA, USA). Sections were then incubated with anti-*MRO* FL-248 antibody (1:500, overnight, 4°C), followed by biotinylated anti-rabbit secondary antibody (1:200, 60 min, RT, ThermoFisher) and streptavidin-HRP/diaminobenzidine (Vectastain ABC Elite peroxidase kit, Vector Laboratories, Burlingame, CA, USA). Hematoxylin was used as a counterstain. Processed sections were examined by acquiring a digital image on the NamoZoomer 2.0RS, (Hamamatsu, Japan) of the entire array and processed using the Metamorph software (Molecular Devices, Sunnyvale, CA, USA).

### Multiplex Western blotting in GC nucleus and cytoplasm

GCs were lysed with NE-PER Nuclear and Cytoplasmic Extraction Reagents (ThermoFisher). Both fractions contained protease inhibitors (Sigma-Aldrich, Oakville, ON, Canada). *MRO* containing plasmid (pSNAP-MRO) was transfected (4ug of DNA, 12hrs; S1 File) into Chinese hamster ovary cells (CHO-K, kindly provided by Dr. Reginald (Department of Immunology, University of Toronto, Toronto, ON, Canada) using Lipofectamine2000 (ThermoFisher). Transfected cells were lysed using the Cell Lytic Mammalian Cell Lysis Kit (Sigma-Aldrich). This served as a positive control for the Western Blot.

For western blotting, 12ug of protein was loaded per lane, resolved on a precast 4–12% Bolt Bis-Tris Plus gradient gel (ThermoFisher) and transferred onto a 0.2 μm nitrocellulose membrane using the Bolt transfer system (ThermoFisher). Membranes were blocked with a 1:1 ratio of Odyssey blocking buffer (LI-COR Biosciences, Lincoln, NB, USA) and 1x TBST (50mM Tris base, 150mM Sodium chloride, 0.05% Tween 20) then incubated overnight at 4°C with rabbit anti-MRO-FL-248 (1:250) (SantaCruz). The mouse monoclonal anti-Actin antibody (1:1000) (Sigma-Aldrich) was used as a loading control and rabbit anti- Histone Deacetylase-1 (HDAC1, 1:500) (Abcam) was used as a nuclear extract specific control. The membranes were then washed 3 times with 1xTBST and incubated with secondary antibodies conjugated to a IRDye fluorophore (LI-COR Biosciences); goat anti-rabbit IgG (800 CW, 1: 10,000) and donkey anti-mouse IgG (680RD, 1:20,000). The membrane was visualized on the Odyssey IR imaging system (LI-COR Biosciences). To ensure the purity of the GCs, protein was extracted as mentioned above from GCs after the initial spin and before and after the Ficoll gradient. Anti-CD45 antibody (Abcam) was used to detect lymphocyte contamination.

## Results

### Tissue-specific expression of *MRO* isoforms

**Fig 2:**
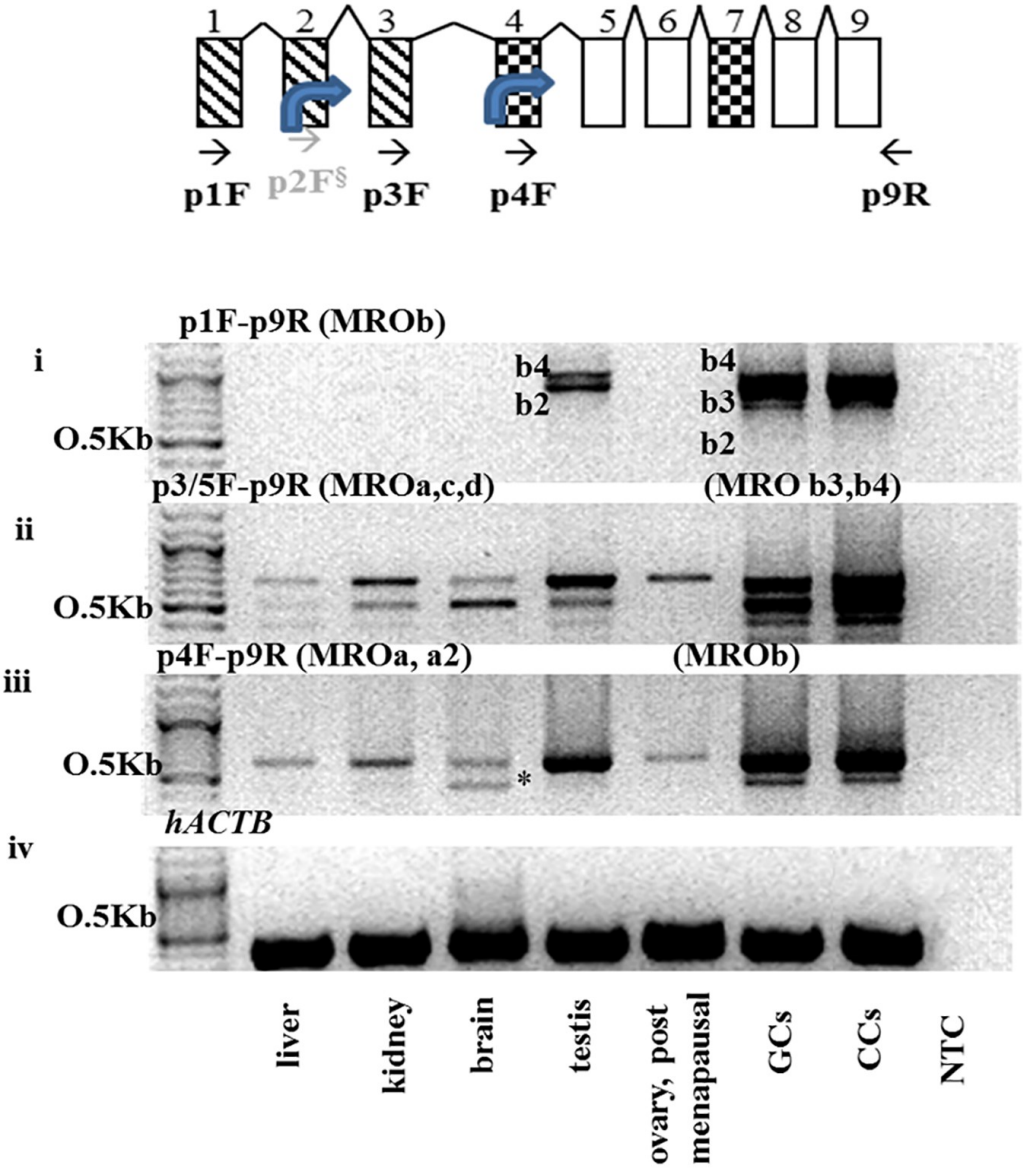
Tissue-specific expression of *MRO* isoforms. RT-PCR using *MRO* primer combinations demonstrated the presence of multiple splice variants, confirming the inferred sequences, and revealing a novel isoform (*MRO*a2). (A) Schematic representation of the *MRO* RT-PCR primer annealing location. (B-i) PCR using primers set p1F-p9R, detected two products in testes and GCs (*MRO*b2 and *MRO*b4). A third isoform, *MRO*b3, was cloned in GCs only. (B-ii) PCR using primers p3/5F-p9R detected *MRO*a variant in the ovary and *MRO*c and *MRO*d in liver, kidney and brain. In the testes and GCs, this primer set detected *MRO*b3 and b4. (B-iii) PCR using primer set p4F-p9R, detected *MROa* (all tissues) and *MROa2* (brain) products. (B-iv) Human actin-B (*hACTB*) was used as loading control. NTC–RNA template control. Cycling conditions were as follows: 3 min at 95°C following by 40 cycles of 95°C/30 sec, annealing at 60°C/ 30 sec and extension at 68°C/ 1–2 min (contingent on the size of the expected amplification product).

Three sets of primers were utilized to clone the *MRO* transcripts. The *MROb (MROb1-b4* transcripts that contain the distal 5’ UTR-promoter were detected by RT-PCR using the p1F-p9R primers (Fig 2A). These PCR products, later confirmed to be *MRO*b2 and *MRO*b4 by sequencing, were found in testes, GCs and CCs (Fig 2B-i), but not in other tissues tested. A third isoform, *MRO*b3, was expressed solely in GCs and CCs. *MRO*b1 was not detected in any cells or tissues. Using primer sets p3/5F-p9R and p4F-p9R –isoforms *MRO*a, *MRO*c and *MRO*d were detected in liver, kidney, brain and whole ovary (post-menopausal) tissues (Fig 2B-ii and 2B-iii).

**Fig 3:**
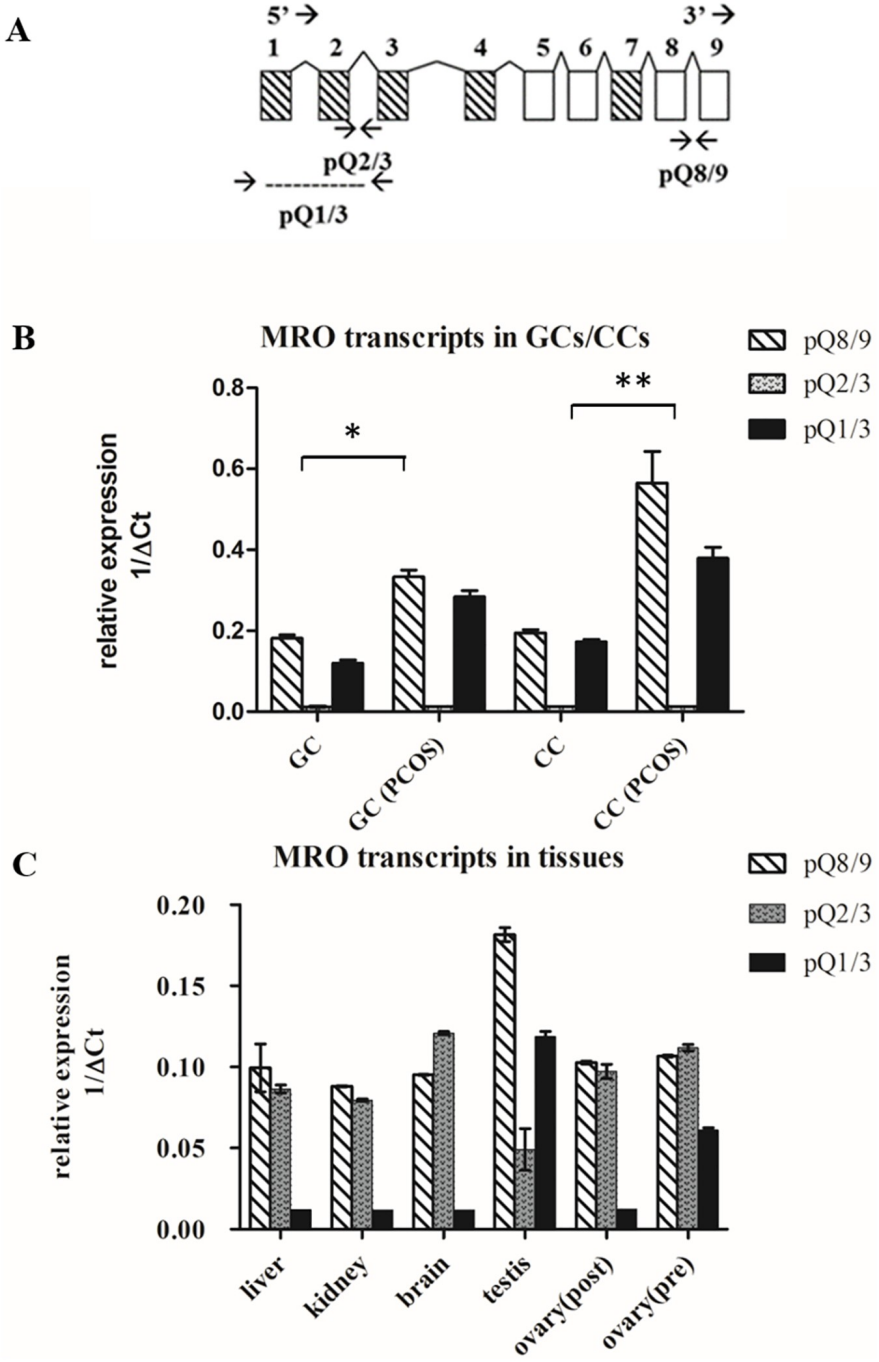
Relative expression of *MRO* transcripts was analyzed by qPCR. (A) Schematic representation of the *MRO* splice variants and the position of the TaqMan probes. Overall expression detected by the pQ8/9 probe. pQ1/3 spans exons 1–3 and detects the 'b' isoforms (*MROb*1-4); pQ2/3 spans exons 2/3 and detects isoforms *MROa*,c,d. The expression of *MRO* was normalized to 18s RNA and presented as relative a expression ratio (1/ΔCt). (B) *MRO* transcripts analysis in GCs and CCs from PCOS and control subjects. The overall expression, shown by exon 8/9 was higher in PCOS cells vs that of controls (* in GCs and * in CCs; p ≤0.05). Inverse expression of exon 1/3 in GCs and CCs vs exon 2/3 was detected. (C) Inverse expression of exon 2/3 vs 1/3 in kidney, liver, brain and post-menopausal ovary.

To better quantify the different *MRO* isoforms, quantitative qPCR was performed using TaqMan gene expression assays. Fig 3A depicts the gene sequence with primers spanning transcript-specific exons. Total *MRO* expression, amplified by pQ8-9 primers set, was evident in GCs and CCs with increased expression (p≤0.05) in PCOS patients (Fig 3B). We also confirmed expression of the distal 5’-UTR-promoter region (amplified by pQ1/3) in these cells. Interestingly, there was little to no expression of the proximal 5’-UTR-promoter (amplified by pQ2/3) in GCs or CCs. In contrast, the proximal 5’-UTR-promoter was found in liver, kidney, post- and pre-menopausal ovarian tissue while the distal 5’-UTR-promoter was absent (Fig 3C).

Our findings, combining standard PCR, TaqMan qPCR, cloning, and sequence analysis results are summarized in Table 4. The results indicate that the distal 5’UTR promoter is uniquely expressed in testicular tissue and within the maturing GCs and CCs of the Graafian follicle. Conversely, the *MRO* transcripts, having the proximal-5’UTR promoter (exon 2 and 3), are expressed in brain, liver, kidney and post-menopausal ovary, and appear to have no role in active folliculogenesis [20].

**Table 4:** Summary of the MRO splice variants detected in GCs, CCs and human tissues by standard PCR and TaqMan qPCR analysis. (+) indicates positive detection and intensity (++++ being the highest). (-) indicates no product detected.

Isoforms	Primer sets	GC/CC	Ovary (post)	Ovary (pre)	Testis	Brain	Liver /Kidney
**MROa, MROa2**	p3/5-p9R, p4R-p9R pQ2/3, pQ8/9	---[	++	++	---	+	+
**MROc**	p3/5-p9R, pQ2/3, pQ8/9	---	---	---	---	++	+
**MROd**	p3/5-p9R, pQ2/3, pQ8/9	---	---	---	---	+	++
**MROb1**	p1F-p9R	---	---	---	---	---	---
**MROb2**	p1F-p9R	+++	---	---	++	---	---
**MROb3**	p1F-p9R, p4R-p9R, pQ1/3	++++	---	+	---	---	---
**MROb4**	p1F-p9R, p4R-p9R, pQ1/3	++++	---	+	++	---	---

### *MRO* protein is localized in GC nuclei

**Fig 4:**
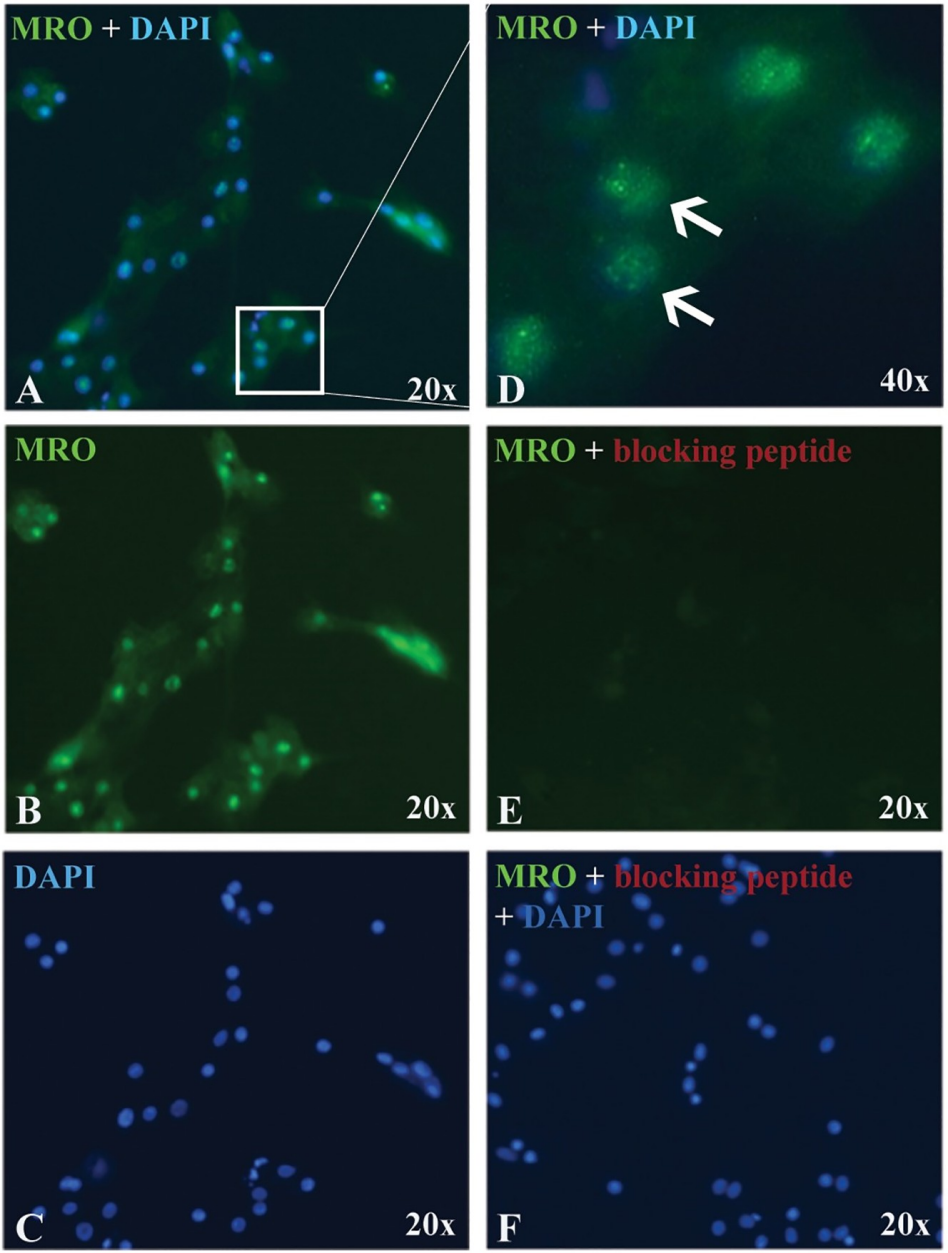
*MRO* expression in GCs. Representative micrograph of GCs immunostained with MRO-FIL antibody (0.4 ug/ml) and counterstained with DAPI (nuclei). (A) Merged signals, (B) *MRO* only, (C) DAPI only. (D) Merged signals under high magnification (x40) micrograph showing *MRO* staining in the nucleus, as well as in the cytoplasm. Antibody specificity was validated with (E) *MRO* blocking peptide (CAT# ab206335). (F) Counterstain with DAPI. All images are taken with the same exposure time.

Next, we determined if *MRO* protein is expressed in luteinized GCs and CCs in culture. using the MRO-FIL antibody, nuclear staining was observed in GCs (Fig 4A–4D) and CCs (S2 Fig). The FL-248 has similar nuclear staining, but also had a high background with this method (data not shown). Using a blocking peptide (CAT# ab206335, Abcam) MRO staining was not evident, indicating that the antibody is specific for the MRO protein (Fig 4E and 4F).

### *MRO* protein detection by immunoblotting

**Fig 5:**
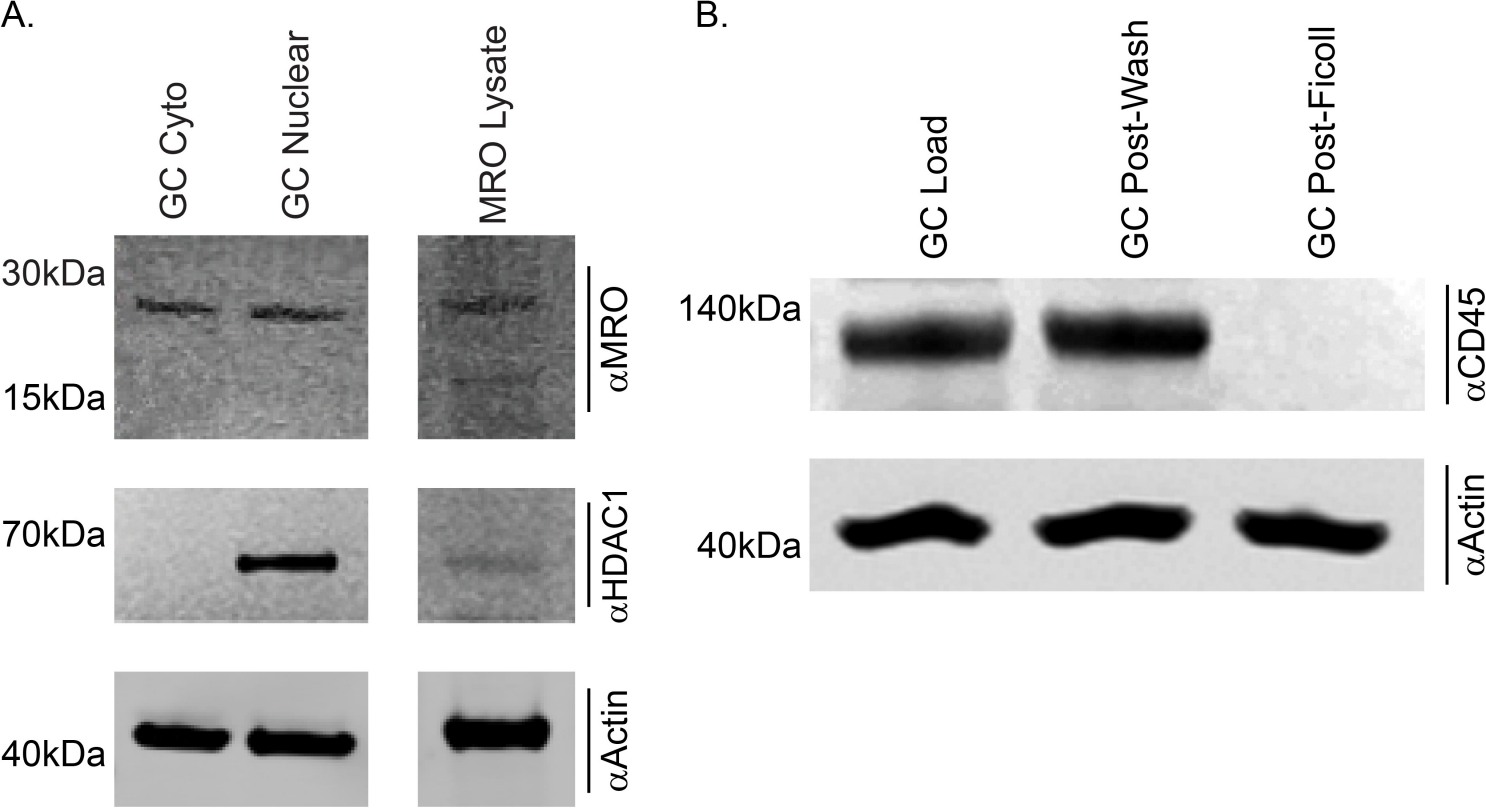
MRO is detected in both the cytoplasm and nucleus by western Immunoblot. A) Nuclear and cytoplasmic extracts were prepared from purified GCs, electrophoresed through a 4–12% reducing SDS-PAGE gel, transferred to 0.2um nitrocellulose, blocked, and probed with the MRO FL-248 (Santa Cruz), anti-HDAC1 (Abcam) and anti-Actin (Sigma-Aldrich) antibodies. MRO (~28kDa) is found at similar abundance in both the cytoplasmic and nuclear fraction (top panel). HDAC1 served as a nuclear specific control to ensure the purity of the extract (middle panel) and Actin served as a loading control (bottom panel). B) Protein extracts from portions of GCs from the different isolation method were resolved. GCs pre-wash (Load), after the 1^st^ wash (post-wash) and after the Ficoll gradient (Post-Ficoll) were probes with the lymphocyte marker anti ἀCD45. Actin served as a loading control (bottom panel).

*MRO* was detected in both the nuclear and cytoplasmic fraction from granulosa cells using the FI-248 antibody (Fig 5A). HDAC1 was detected in only the nuclear fraction, confirming that this fraction contains nuclear derived proteins and that the cytoplasm fraction did not contain any contaminating nuclear deprived protein. As indicated by the absence of CD45 positive signal, the GCs were purified after the Ficoll gradient and did not contain any lymphocytes (Fig 5B). Anti-actin was used as a loading control.

### *MRO* protein is detected in active granulosa cell layers in human ovarian tissue sections in non-PCOS and PCOS ovaries

**Fig 6:**
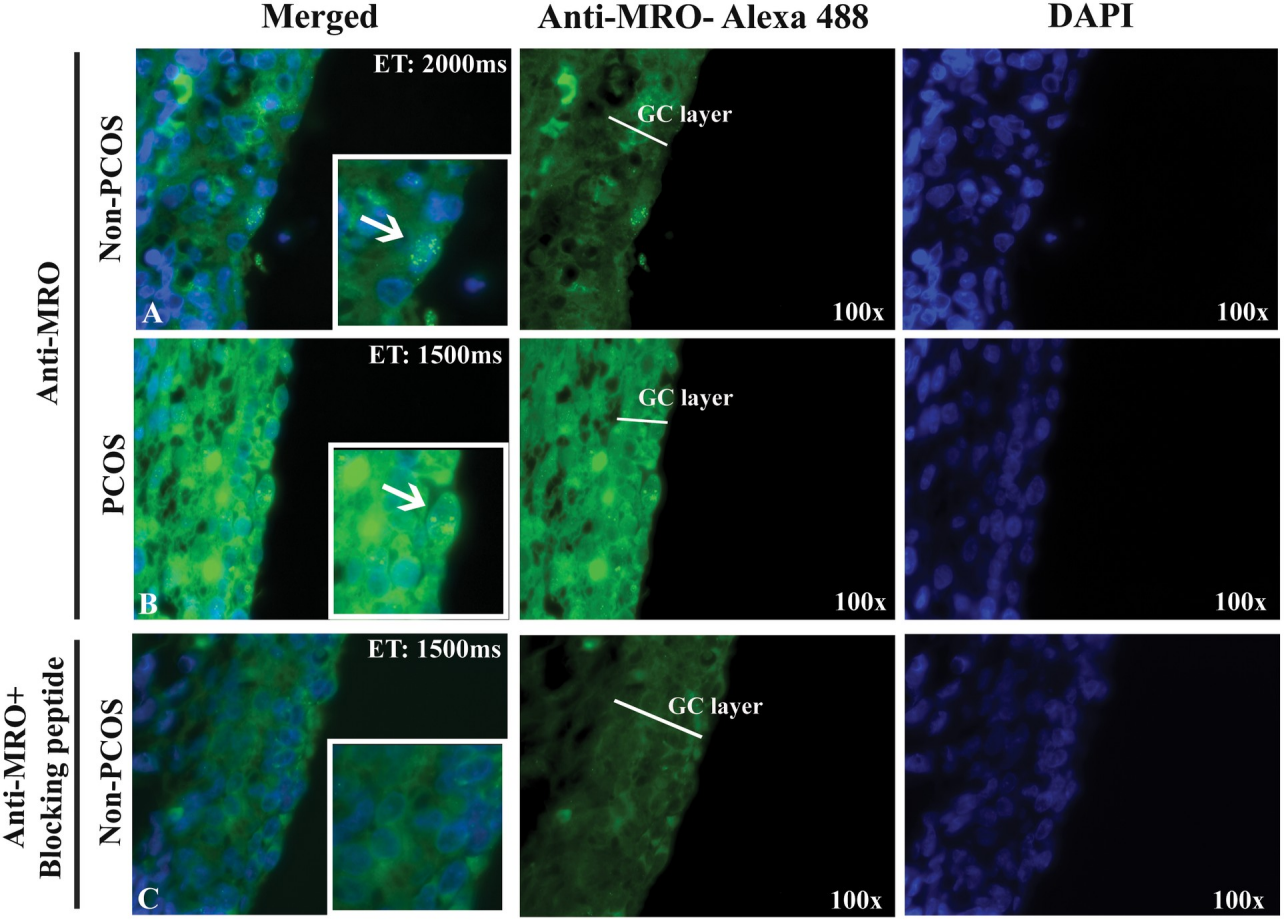
*MRO* expression in human ovarian sections. Immunofluorescence was performed on non-PCOS (A) and PCOS (B and C) ovarian sections using *the* FL-248 antibody (4ug/ml). Nuclear staining is apparent in the granulosa cells (magnified images), while the blocking peptide abolished the MRO signal (C). *MRO* expression was observed in green (Alexa-fluor 488) and nucleus in blue (DAPI). Magnification: x100. Duration of exposure: 2000 milliseconds (non-PCOS) and 1500 milliseconds (PCOS) demonstrating that even with short exposure the fluorescence intensity is higher in PCOS samples.

To compare *MRO* protein expression in PCOS and non-PCOS, ovarian tissue sections were immunostained using the FL-248 antibody, as it gave superior results over the FIL-Ab used for ICC. Similar cytoplasmic and nuclear staining was observed in both the PCOS and non-PCOS ovarian tissue sections, although higher cytoplasmic background staining is observed in the PCOS section (Fig 6). Blocking peptide abolished the specific nuclear staining and produced much less background staining, suggesting that the cytoplasmic staining might be, impart due to non-specific targets.

**Fig 7:**
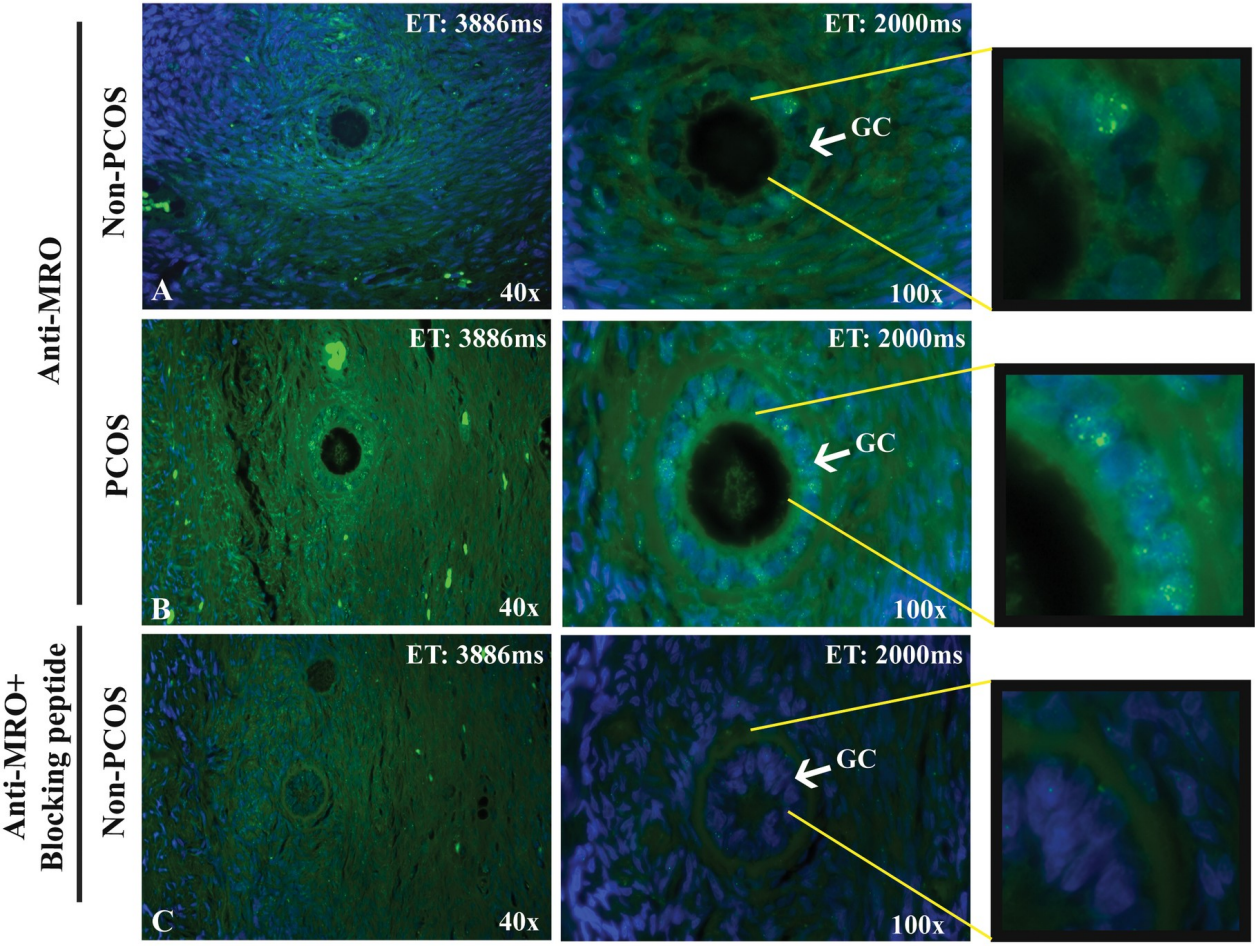
*MRO* protein expression in human primary follicle. Immunofluorescence was performed on an ovarian section from a non-PCOS (A) and a PCOS (B) patients using the FL-248 antibody (4ug/ml). Blocking peptide in (C) demonstrated the reduced fluorescence signal in the tissue. *MRO* protein is present in GCs and in some stromal cells. *MRO* expression is indicated in green (Alexa-fluor 488) and nucleus in blue (DAPI). Magnification: x4 and x100; Duration of exposure in 40x: 3886 and 100x: 2000 milliseconds.

The expression of *MRO* protein was also evident in human primary follicle (Fig 7), with similar pattern of nuclear staining in GCs and CCs and high cytoplasmic background staining in some stromal cells. A stronger staining was observed in PCOS tissues and the nuclear staining in GCs, which was abolished in the presence of the blocking peptide (Fig 7C).

### *Minute MRO* protein expression in human ovary and testis tissues

**Fig 8:**
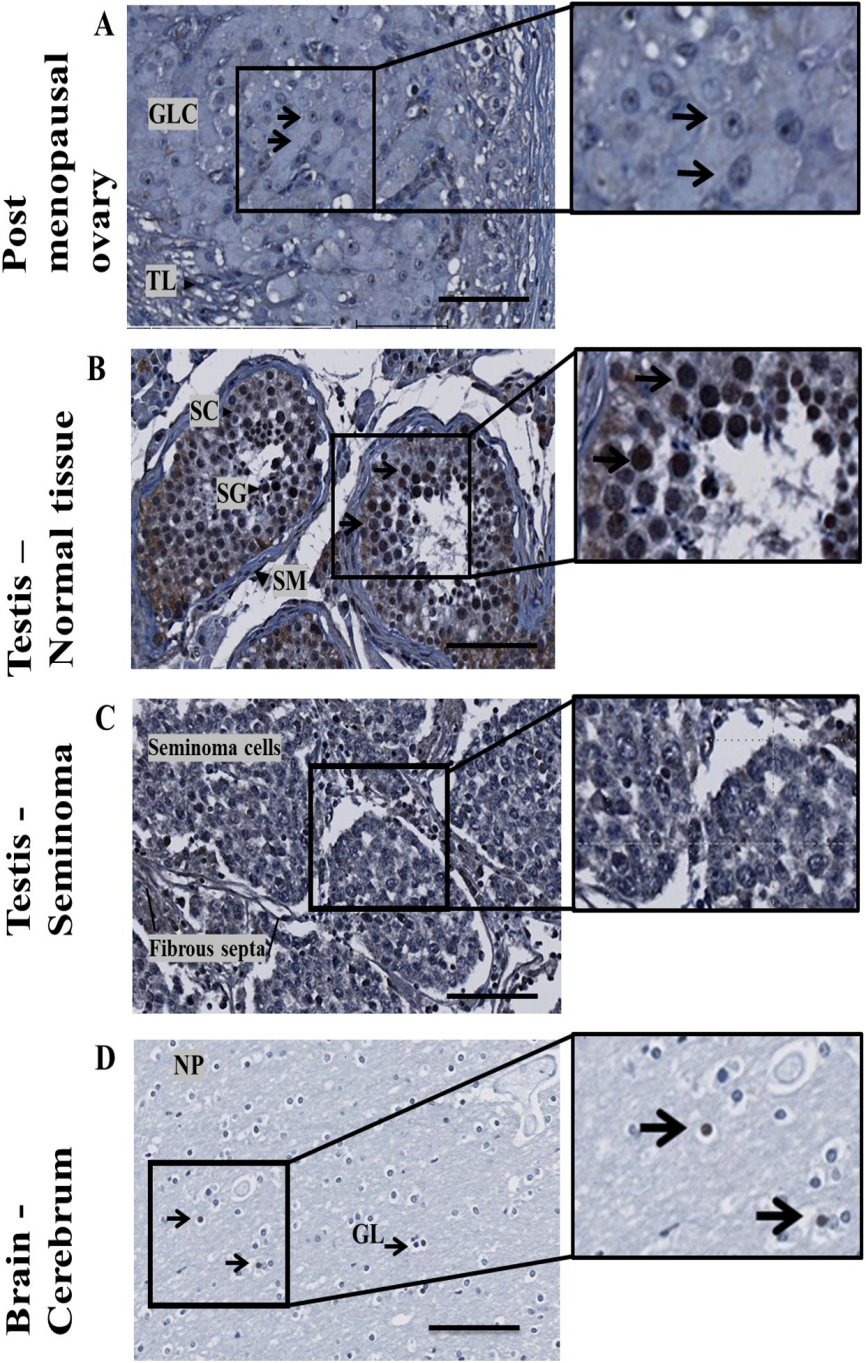
*MRO* expression in human tissues with FL-248. *MRO* expression in (A) ovarian tissue of a post-menopausal subject with **no active folliculogenesis** (age 52); (B) adult normal testis; (C) Typical testicular seminoma; (D) Cerebrum (brain) tissue. Abbreviations: GLC–granulosa-lutein cells, TL–theca lutein cells, SC–Sertoli cells, SG–spermatogonia, PS–primary spermatocyte, SP–spermatids, NP–Neuropil, GL–glial cells. Bar ruler, 100um.

The expression of *MRO* protein in ovarian tissue of a post-menopausal subject (age 52), testis and brain was determined (Fig 8). Nuclear staining was evident in sparse luteal granulsoa cells (GLC) (Fig 8A) and in spermatogonia cells in adult testis (Fig 8B) but absent in testicular seminoma–tissue displaying minimal spermatogenesis (Fig 8C). Nuclear staining was occasionally evident in glial cells from the cerebrum (brain) tissue (Fig 8D).

### MRO antibodies

We have tested the all available commercial and in-house MRO antibodies in combination with transfected lysates and recombinant proteins and peptides (S1 Table and S2 Table). While all could detect the cell-free *in-vitro* MRO product, the antibodies detect mainly high molecular weight nonspecific products, while no detection or a very faint detection was observed for the MRO (S3 Fig)

## Discussion

Herein, we report for the first time the tissue-specific expression pattern of the human *MRO* gene and protein. We have demonstrated that 1) transcripts derived from distal 5’UTR promoter region was selectively and exclusively expressed in luteinized GCs, CCs and in testicular germ cells. 2) The transcript from the proximal 5’UTR promoter was found in the brain, kidney, liver, and whole postmenopausal ovary tissues. 3) We also confirmed earlier results of *MRO* overexpression in GCs and CCs from PCOS patients. 4) Nuclear staining of the *MRO* protein was observed in GC and CCs, with weak staining of the cytoplasm, supporting previous results from Smith et al. [2]. 5) Last, in light of the limited information in the literature regarding the *MRO* protein and antibodies, we provide a detailed list of antibodies that are available and their inaccuracies, suggesting caution be exercised in interpreting findings arising from their usage.

Following our hypothesis that MROb isoforms (containing the distal exon 1) is expressed only in GCs and CCs from the follicle retrieved from super-ovulated subjects, it is not surprising that the whole ovary–a tissue that contain only few secondary follicles in normal physiological state or mainly theca and stroma tissues in post-menopausal ovaries, show very low or no expression of MROb isoforms. This is supported by the immunostaining of human ovaries from post-menopausal ovaries showing only disperse MRO staining. It is possible that the different 5’-UTR of the *MRO* in GCs and CCs cells and in spermatocyte/spermatid reflects their functional role in the human reproductive system. Alternative mRNA splicing and differential promoter utilization could determine tissue-specific gene expression [21, 22], thereby allowing multiple regulatory pathways be differentially regulated in a cell-specific manner [23–25]. Splicing mechanisms are important in development, ageing and are altered in various pathological conditions [26, 27]. Genes regulated by hormones are known to have distinct promoters [28–30]. For example, the gene for the follicle stimulating hormone receptor (*FSHR*) has a remarkable cell-specific expression that is achieved by exon-skipping and regulatory elements that lie far from the transcription start site [31]. A differential tissue expression profile has also been reported for the human sex hormone binding globulin (*SHBG*) [32] and Cytochrome P450 Family 19 Subfamily A Member 1 *(CYP19A1*, aromatase P450) [33]. The 5'- UTR of CYP19A1 transcripts in gonads, placenta, and brain are encoded by different tissue-specific first exons, which are alternatively spliced onto a common site just upstream of the translation start codon. This is remarkably similar to the alternative splicing of the *MRO* transcript we have profiled in this study. The biological role of *MRO* is unclear. Due to the HEAT domain, it is a candidate transcription factor and may have a role in regulating gene expression. This gene expression may be different depending on the isoform of *MRO* being expressed.

The murine *Mro* transcript was first discovered in murine embryos during male gonadal somatic cell development, but was not detected in female gonads at this developmental stage [2]. Although both male and female knockout mice are viable and fertile [11], no detailed studies have been reported on the adult female mice. This lack of evidence for *Mro* expression in the mouse fetal female gonads may be due to a temporal expression of *MRO* as we demonstrate in humans (loss of expression in postmenopausal women). Time course studies on gene expression in mouse cumulus oocyte complex around the peri-ovulatory period reveal that *Mro* is down regulated 8 h after the administration of hCG [34]. In addition, Fan et al [35] reported that the MRO was identified together with a group of genes regulated by Erk1/2 in GCs downstream of gonadotropin signalling [35]. In S1 Table of the aforementioned paper, the authors reported that *MRO* expression was decreased 9-fold in wild-type GCs after hCG treatment. Interestingly, a 6-fold increase was observed in conditionally inactivated *Erk1/2*^-/-^ GCs. Other genes that were similarly down-regulated included *Cyp19a1*, *Cyp17a1* and *Fshr—*all are well known to be involved in folliculogenesis. This could suggest that these genes and *MRO* act downstream of gonadotropin and *Erk1/2* signaling pathway. In contrast to wild-type mouse *Mro*, we observed high expression of the human *MRO* gene in luteinized GCs. Importantly, we could not detect any *MRO* protein in mouse or rat tissue using any *MRO* antibodies (data not shown), which limit us from further exploring the biological function of the protein in a rodent model.

The *MRO* protein family (*MRO*-like) are newly discovered proteins with no known function to date. The lack of information regarding the characteristics the *MRO* protein (domains, secondary structure, cellular localization etc.) made it challenging to detect the native protein. However, we were able to successfully detect the native protein from both the nuclear and cytoplasmic fractions, using immunoblotting. Although we loaded in excessive amount of protein, the signal was very faint. This is probably due to several factors including very low protein expression in these cells and antibodies that were raised against either a peptide or an unmodified version of the protein. Taken together, these results suggest that, these antibodies should not be used for immunoprecipitation of the *MRO* protein in future studies.

To this end, we investigate the possibility that *MRO* has intrinsically disordered proteins regions (IDPs). Using the PONDR algorithms (http://www.pondr.com/) and RaptorX (http://raptorx.uchicago.edu/), we predicted that the *MRO* has several unstable *IDP* regions. IDP regions fail to form a stable structure, yet they exhibit biological activities [36, 37]. IDPs often function as a hub of interaction between multiple binding partners in a signal transduction cascade or in the regulation of transcription through the binding or recruitment of other transcription factors [38–40].

In conclusion, tissue-specific transcriptional variants of the *MRO* gene are uniquely expressed in human luteinized GCs and in the human testis. Future investigations, based on the tissue specific expression groundwork we report here, should reveal the significance of the *MRO* gene in normal human gonadal physiology, and potentially provide further insight into the pathophysiology of PCOS and/or other conditions leading to male or female sub-fertility. Due to the close proximity of GCs, CCs and the oocyte, the variation in *MRO* may have a direct impact on follicular development and oocyte maturation. However, more studies investigating the biological function or protein-protein interactions of *MRO* with some unknown binding partners in both the mouse/rat models and in human samples would be required to validate these claims.

## Supporting information

S1 Fig Detailed Sequence alignment of the MRO transcripts, deduced protein isoforms and antibodies position. (TIF)

S2 Fig *MRO* expression in CCs. Representative micrograph of CCs immunostained with MRO-FIL antibody (0.4 ug/ml) and counterstained with DAPI (nuclei), as mentioned in Fig 4. (A) *MRO* only, (B) Merged signals. (C) Antibody specificity was validated with (E) *MRO* blocking peptide (ab206335). (F) Counterstain with DAPI. All images are taken with the same exposure time.
(TIF)

S3 Fig Recombinant MRO detection in transfected lysates and in-vitro product. Representative multiplex Western blotting analysis of recombinant MRO. The gel was loaded with MRO transfected CHO-cells lysates (4ug). Membranes were blotted with the AER-Ab (green signal) and MGMT (red signal). pSNAP-MRO vectors (pSNAP -1, 2 and 3), control vector or mock transfection and the cell-free MRO product (Ivt-MRO).
(TIF)

S1 Table MRO antibodies. List of all available primary antibodies tested for the detection MRO in immunoblots and immunohistochemistry in this study. Antibodies, peptides, and protein lysates used in this study (S1 Table and S2 Table) were obtained from Abcam (Toronto, ON, Canada), Santa Cruz Biotechnology (Dallas, Texas, USA), Aviva Systems Biology (San Diego, CA, USA), Sigma-Aldrich (Oakville, ON, Canada), Novus Biologicals (Oakville, ON, Canada), and OriGene Technologies (Rockville, MD, USA). We also designed and generated an affinity purified polyclonal antibody as raised in a rabbit against a deduced peptide sequence from exon 6 (Genescript, Piscataway, NJ) and was labeled MRO-AER. All antibodies were raised in rabbit against a peptide sequence or full-length protein (prepared in non-mammalian wheat germ system). The A peptide competition assay (PCA) was performed to confirm the specificity and reactivity of the peptide antibody and overexpressed lysates of variant 1 and 2 *MRO* clones were used as positive controls. Detection of *MRO* by immunoblotting in transfected cell lysate and from in-vitro, cell free expression system in shown S2 Fig.
(DOCX)

S2 Table MRO recombinant proteins and transfected lysates. List of all available blocking peptides, proteins, and transfected lysates for the detection MRO in immunoblots and immunohistochemistry in this study.
(DOCX)

S1 File Recombinant *MRO* protein expression methods. (DOCX)
