# Supplementary figures and images for: The elusive MAESTRO gene: Its human reproductive tissue-specific expression pattern
Source: PLoS One. 2017 Apr 13;12(4):e0174873. doi: 10.1371/journal.pone.0174873 (PMC5391009; doi:10.1371/journal.pone.0174873)

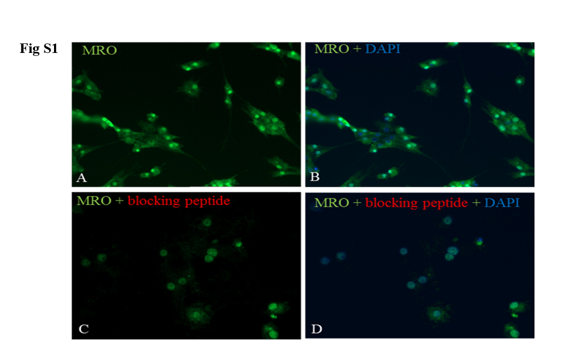

Supplement: S1 Fig — (TIF) [file pone.0174873.s001.tif]

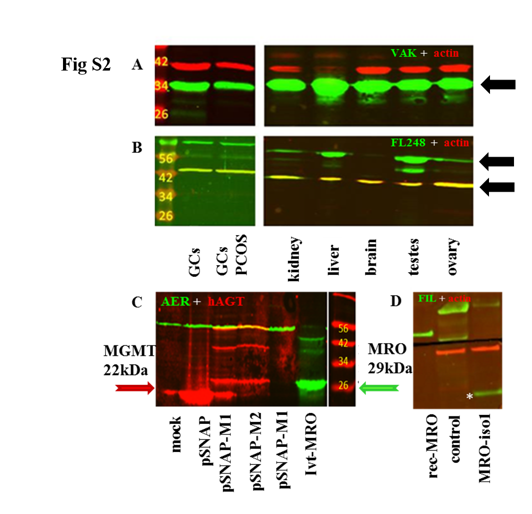

Supplement: S2 Fig — Representative micrograph of CCs immunostained with MRO-FIL antibody (0.4 ug/ml) and counterstained with DAPI (nuclei), as mentioned in Fig 4. (A) MRO only, (B) Merged signals. (C) Antibody specificity was validated with (E) MRO blocking peptide (ab206335). (F) Counterstain with DAPI. All images are taken with the same exposure time. (TIF) [file pone.0174873.s002.tif]

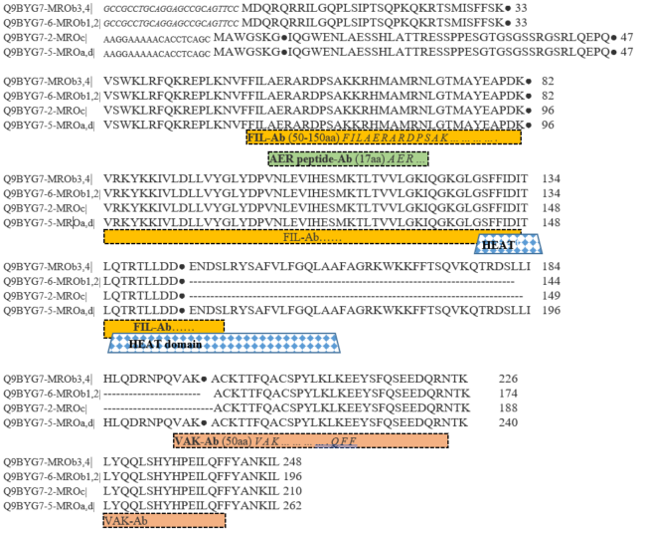

Supplement: S3 Fig — Representative multiplex Western blotting analysis of recombinant MRO. The gel was loaded with MRO transfected CHO-cells lysates (4ug). Membranes were blotted with the AER-Ab (green signal) and MGMT (red signal). pSNAP-MRO vectors (pSNAP -1, 2 and 3), control vector or mock transfection and the cell-free MRO product (Ivt-MRO). (TIF) [file pone.0174873.s003.tif]
