# Supplementary material for: The elusive MAESTRO gene: Its human reproductive tissue-specific expression pattern
Source: PLoS One. 2017 Apr 13;12(4):e0174873. doi: 10.1371/journal.pone.0174873 (PMC5391009; doi:10.1371/journal.pone.0174873)
Supplement: S1 Table — List of all available primary antibodies tested for the detection MRO in immunoblots and immunohistochemistry in this study. Antibodies, peptides, and protein lysates used in this study (S1 Table and S2 Table) were obtained from Abcam (Toronto, ON, Canada), Santa Cruz Biotechnology (Dallas, Texas, USA), Aviva Systems Biology (San Diego, CA, USA), Sigma-Aldrich (Oakville, ON, Canada), Novus Biologicals (Oakville, ON, Canada), and OriGene Technologies (Rockville, MD, USA). We also designed and generated an affinity purified polyclonal antibody as raised in a rabbit against a deduced peptide sequence from exon 6 (Genescript, Piscataway, NJ) and was labeled MRO-AER. All antibodies were raised in rabbit against a peptide sequence or full-length protein (prepared in non-mammalian wheat germ system). The A peptide competition assay (PCA) was performed to confirm the specificity and reactivity of the peptide antibody and overexpressed lysates of variant 1 and 2 MRO clones were used as positive controls. Detection of MRO by immunoblotting in transfected cell lysate and from in-vitro, cell free expression system in shown S2 Fig. (DOCX) [file pone.0174873.s004.docx]

| **Antibody Name** | **company** | **cat. #** | **Antigen** | **Details** | **Application** | **Company reference** | **WB results (kDa)** | **ICC/IHC Results** |
| --- | --- | --- | --- | --- | --- | --- | --- | --- |
| **FIL** | Abcam | ab181048 | Synthetic peptide within 50-150aa. | Protein A purified, monoclonal | WB, IP | Human ovary cancer lysate, Human fetal brain, heart lysate | 29, 36 | Nuclear, cytoplasmic |
| **FL-248** | SCBT | sc-134943 | full length protein (1-248aa) produced in *E.coli*, | Protein A purified, polyclonal | WB, ICC | Jurkat and HeLa whole cell lysates (showing only the 30bp band). | 29 (faint), 36, 72 | Nuclear, cytoplasmic |
| **AER** | Designed/produced by GeneScript |  | synthetic peptide, 17aa from exon 6 | Protein A purified, monoclonal | n/a | n/a | 29 (faint), 36 | Nuclear, cytoplasmic |
| **DDE** | SIGMA | HPA022011 | full length protein (1-248aa), epitope signature tag (PrEST) | Affinity purified, polyclonal | WB, IHC | Strong cytoplasmic positivity in human cerebellum Purkinje cells. Over-expression lysate (C-terminal myc-DDK tag detected by anti-DDK in HEK293T cells) | 36, 72 | strong cytoplasmic |
| **SFF** | Abcam,  Identical to DDE antibody (Sigma) | ab150813 | synthetic peptide within 142-248aa | Affinity isolated, polyclonal | WB, IHC | A 30kDa band in WB with overexpressed lysate. Protein tag was detected by anti-DDK, not by anti MRO. | 36, 72 | cytoplasmic |
| **VAK** | Aviva | ARP60588 | Synthetic peptide toward C-terminal | affinity purified, polyclonal | WB | WB Positive Control: Jurkat cell lysate. Very faint band at 30 and 100kDa | 36, 72 | no detection |
| **T-15** | SCBT | sc-85046 | epitope within an internal region of the protein | polyclonal | No information |  | 42, 72 | non specific cytoplasmic |
