# Supplementary material for: The elusive MAESTRO gene: Its human reproductive tissue-specific expression pattern
Source: PLoS One. 2017 Apr 13;12(4):e0174873. doi: 10.1371/journal.pone.0174873 (PMC5391009; doi:10.1371/journal.pone.0174873)
Supplement: S2 Table — List of all available blocking peptides, proteins, and transfected lysates for the detection MRO in immunoblots and immunohistochemistry in this study. (DOCX) [file pone.0174873.s005.docx]

| **Protein/over expressed lysate** | **company** | **cat. #** | **Antigen** | **controls (company website only)** |
| --- | --- | --- | --- | --- |
| Blocking peptide for Anti-MRO antibody [EPR12158] | Abcam | ab206335 | Recombinant, no further information |  |
| Blocking Peptide (the C terminal of MRO) | Aviva | AAP60588 | Synthetic peptide against the VAC abtibody | Synthetic peptide designed for use in combination with anti-MRO Antibody (ARP60588_P050) |
| Recombinant Human MRO protein | Abcam | ab164453 | Recombinant, full length produced in Wheat Germ System, proprietary tag N-Terminus | 50KDa on a 12.5% SDS-PAGE stained with Coomassie Blue |
| MRO (Human) Recombinant Protein (P01) | Novus | H00083876-P01 | Recombinant, full length produced in Wheat Germ System, proprietary tag N-Terminus Human MRO full-length ORF (NP_114145.1 1-248aa.) | 50KDa on a 12.5% SDS-PAGE stained with Coomassie Blue |
| MRO Overexpression Lysate (Native) | Novus | NBL1-13231 | The protein contains a C-terminal DDK tag, transcript variant d | 38KDa on a 12.5% SDS-PAGE stained with Coomassie Blue |
| Transient overexpression lysate of MRO, | Origene | RC225220 | MRO (Myc-DDK-tagged)-Human maestro (MRO), transcript variant 2 | WB Positive Control: Jurkat cell lysate. Very faint band at 30 and 100kDa |
