# Supplementary material for: The elusive MAESTRO gene: Its human reproductive tissue-specific expression pattern
Source: PLoS One. 2017 Apr 13;12(4):e0174873. doi: 10.1371/journal.pone.0174873 (PMC5391009; doi:10.1371/journal.pone.0174873)
Supplement: S1 File — (DOCX) [file pone.0174873.s006.docx]

**Appendix S1 – Recombinant *MRO* protein expression methods**

**Cloning into pGEM-Teasy vector**

PCR reactions containing the various *MRO* isoforms were cleaned with MinElute Reaction Cleanup Kit (Qiagen), and were cloned into the pGEM-Teasy expression vector (New England Biolab (NEB), Whitby, ON, Canada) and transformed into DH5ἀ competent cells (NEB). The vector was for IPTG-induced bacterial expression. The QIAprep Spin Miniprep kit (Qiagen) was used to isolate plasmids before sequencing.

**Subcloning into pSNAP^TM^ vector**

The pSNAP**^TM^** expression vector (NEB) was used for expression in mammalian cell lines. SNAP epitope-tagged isoforms containing the open reading frame (ORF) of the *MRO* gene from exons 4 to 9 or 6 to 9 were subcloned from the pGEM-Teasy vectors (pSNAP-1, pSNAP-2, respectively). For additional tagging a pSNAP vector containing *MRO* exon 4-9 and a 6-histidine c-terminal tag was constructed (pSNAP-3). The SNAP-tag was 20 kDa and detectable by the mouse anti-O6-Methylguanine-DNA Methyltransferase (MGMT) antibody (Millipore, Billerica, MA, USA). All constructed vectors were sequenced to confirm the existence of an ORF for protein expression.

***Transfection into CHO-K cells***

Chinese hamster ovary cells (CHO-K, a gift from Dr. Reginald Gorczynski) cells were transfected using Lipofectamine2000 (Thermo Fisher) with 4 ug of DNA. After 12 h, the cells were washed and lysed with Cell Lytic Mammalian Cell Lysis Kit (GE Healthcare, Piscataway, NJ).

**Multiplex Western Blotting**

The PureExpress™ cell-free protein expression system (NEB) was used to create *in vitro* *MRO* protein (ivt-MRO) according to the manufacturer’s instruction. Transfected CHO-K with the pSNAP-MRO vectors were lysed with the Protein Extraction Kit buffer containing protease inhibitors (PRO-PREP^(TM)^ iNtRON , Korea) and For Western blotting, 4 ug of protein was loaded per lane on a gel and was immunoblot as mentioned in the main manuscript (S3 Fig.). Each membrane was co-incubated with the MRO antibody (1:250-500). For loading control the MGMT antibody (1:1,000, Millipore) was used for pSNAP transfected CHO-K cell line. The membranes were then incubated (30 min, RT) with secondary antibodies conjugated to IRDye fluorophores (LI-COR Biosciences), the goat anti-rabbit IgG (800 CW, green signal, 1:10,000) and goat anti-mouse IgG (680 RD, red signal, 1:15,000). The membranes were visualized on the Odyssey IR imaging system (LI-COR Biosciences).
